# New ways in critical management and organization studies: Honneth and the method of normative reconstruction

**DOI:** 10.3389/fsoc.2025.1646745

**Published:** 2025-08-08

**Authors:** Max Visser

**Affiliations:** Institute for Management Research, Radboud Universiteit, Nijmegen, Netherlands

**Keywords:** Axel Honneth, recognition theory, normative reconstruction, critical theory, CMS

## Abstract

While current global ecological, social and cultural challenges and the role of organizations therein call for involvement of critical management studies, critical scholars have been divided over how to engage with organizational practice and practitioners. In order to move this issue forward, the purpose of this paper is to propose a new approach to critical analysis in the form of the method of normative reconstruction of the Frankfurt social philosopher Axel Honneth. Toward that purpose, the basic tenets of this method of normative reconstruction are discussed and empirically illustrated through two cases, while the paper ends with conclusions and discussion.

## 1 Introduction

To say that we currently live in a period of major global challenges may be a gross understatement. As I write this in the Summer of 2025, climate and ecological problems—evidenced by rampant wildfires, record floods, many “storms of the century” and record temperatures in nearly every part of the world—are growing in number and scope, with no viable systemic solutions proposed or enacted yet. Armies clash into one another in various parts of the world, throwing supply chains in disarray. The accuracy and credibility of information shared in nearly every media modality is under question, thanks in large part to the influence of privatized media platforms that give voice to purveyors of misinformation. Accompanied by the sudden, chaotic and brutal turn to authoritarianism and isolationism in the United States, neoliberal capitalism and financialization still reign as the prevailing ideology justifying exploitive practices against nature and people, held supreme under the guise of the purportedly innate human values of individual and market freedom.

In all of these challenges, the role of organizations is crucial. They are causes of these challenges (e.g., fossil fuel corporations, arms manufacturers, private equity firms, technology monopolies, investment banks, tax and management consultancy firms, armies, militias), part of possible solutions (e.g., public sector organizations, NGO’s, international treaty organizations, labor unions, social enterprises, political and social activist groups, cooperatives), or a bit of both (e.g., pharmaceutical firms, U. N. task forces, religious groups, public-private initiatives, educational institutions, think tanks) (Adler, 2022; Ferreras et al., 2022).

This shared responsibility (for better or worse) for global challenges necessitates new organizational forms and ideas that diminish organizations’ ecological footprint, financial and fiscal irresponsibility and oppressive management and work practices. This should be a major concern for management and organization scholars in general and to critical management scholars in particular. Critical management studies (hereafter CMS) traditionally has thought most deeply and critically about organizations and their role in society (e.g., Adler et al., 2008; Alvesson and Spicer, 2025; Scherer, 2009), and thus seems best positioned to take the lead in tackling these global challenges.

However, the concrete stance of CMS scholars toward organizations, practitioners and their problems is far from equivocal and in transition. For a long time (and in spite of pressing global challenges), they have tended to disengage from practice, fearing that practitioners may colonize, distort or pervert their ideas (Fournier and Grey, 2000; Visser, 2010). Only in the past decade, these scholars have fully started to think about how and where to engage with practice, leading to views of CMS as critically performative (Alvesson and Spicer, 2012; Spicer et al., 2009, 2016). While the exact nature of these forms of performativity gave rise to a rather intense and inconclusive theoretical debate, other CMS scholars did not wait for the outcome thereof and actively engaged with practice, mostly through participatory action research in cooperatives and alternative organizations (e.g., Just et al., 2021; Leca and Barin-Cruz, 2021).

Still others deplored “the idea of transforming CMS in some form of action research or critical consultancy” (Spoelstra and Svensson, 2016, p. 74), and, alternatively, suggested involvement as CMS scholars in practical situations in which they can make a real difference (e.g., by becoming active in a “reflective insider” role within their own universities and business schools and/or by applying critical pedagogy in their own teaching), coupled with a “distant outsider” role toward practical situations in which making a real difference is much more difficult (e.g., in engaging with powerful vested corporate and financial interests) (Fleming and Banerjee, 2016; Visser, 2019).

However, both roles have met with difficulties and constraints. Regarding the “reflective insider” role, this has by and large not been taken up very extensively. Even though in the past decennia universities and business schools, influenced by neoliberal policy doctrines like New Public Management, have become more “business-like” and technocratic, managing academic performance on the basis of systems of “targets and terror” (Jones et al., 2020; Van Houtum and Van Uden, 2022; Visser et al., 2024), critical engagement of in particular senior CMS scholars with these developments has been described as less fanatical, even cursory (Butler and Spoelstra, 2014; Parker, 2023), even when junior CMS scholars were disproportionally affected by oppressive “micro terror” measures (Ratle et al., 2020).

Regarding the “distant outsider” role, it has been noted that CMS scholars who even marginally engage with corporate settings soon run into (sometimes insoluble) dilemmas and tensions between critical “ideal-typical criteria, such as cooperative production, worker self-management, and solidarity” on the one hand, and practitioner demands, corporate power and institutional pressures on the other (Zanoni et al., 2017, p. 583; Butler et al., 2018). Even alternative organizations, which already seem to share some critical organization ideals and to which CMS scholars often are less “distant” and “outside,” may turn out to be hesitant, as the experience of King and Land (2018) with the organization that democratically rejected internal democracy attests.

These issues, however, are not new or unique to current-day CMS. Already 80 years ago Max Horkheimer, one of the founders of the Frankfurt school of critical theory, sketched the necessity of social scientists “to take an active part in the direction of the social life of humanity” (Horkheimer, 1937, p. 292), and to do so without accepting the espoused values of institutions and organizations at face value *and* without positing normative ideals and dogma’s that would somehow appear to be beyond a critical analysis themselves. This may be accomplished by “relating social institutions and activities to the values they *themselves* set forth as their standards and ideals. Thus, the activities of a political party may be investigated in the light of the avowed aims and ends of the party without accepting these as valid or evident. If subjected to such an analysis, the social agencies most representative of the present pattern of society will disclose a *pervasive discrepancy* between what they actually are and the values they accept,” which analysis in its turn would “force the categories of social theory to become critical” (Horkheimer, 1941, p. 122; italics mine).

An approach to CMS’ relations to practice along these lines would solve some of its current predicaments. First, such an analysis of “pervasive discrepancies” permits a critical engagement with organizational practices from both a “reflective insider” and a “distant outsider” position (Visser, 2019). Second, such an analysis is in principle compatible with more specific current CMS methodologies, from participative action research and ethnographic inquiry that are “inside and close” to more detached forms of social science or philosophical inquiry that are “outside and distant” (Spoelstra and Svensson, 2016, p. 76; Duberly and Johnson, 2009). Third, such an analysis absolves the CMS researcher from the responsibility to bring in ideals from the outside and instead, in an immanent fashion, takes the espoused ideals and values of the organization itself as point of departure (Zanoni et al., 2017).

In the light of these debates, I propose a new immanent approach to critical analysis in the form of the theory of recognition and method of normative reconstruction of Axel Honneth, a contemporary German social philosopher in the Frankfurt School tradition of Horkheimer, Adorno, Marcuse, Habermas and others (Anderson, 2011; Jütten, 2015; Zurn, 2015). More specifically, the purpose of this paper is to show how this theory and method may supplement existing CMS theory and engagement with practice.

In this way, the paper makes two contributions. First, while already making significant inroads in management and organization studies (MOS) (see recently Hancock, 2024; Honneth, 2023; Mao and Xue, 2022; Rostain and Clarke, 2025), Honneth’s ideas “have yet to be widely taken up by critical students of management,” and thus build on the Frankfurt heritage of earlier CMS theory (Alvesson and Willmott, 2012, p. 261; Scherer, 2009). Next to Willmott (2020), this paper represents a novel attempt in that direction. Second, while normative reconstruction as a method has been applied to a variety of institutional spheres and economic practices (Helm and Seubert, 2020; Hartmann and Honneth, 2006; Kristensen and Kristensen, 2023; Sutterlüty, 2017; Visser and Arnold, 2022), this paper represents a novel application at the organizational level.

Toward that purpose and these contributions, in the second section of this paper the basic tenets of Honneth’s method of normative reconstruction and recognition theory are discussed. In the third section, this method is empirically illustrated through two case examples from the literature, while the paper ends with conclusions and discussion.

## 2 Normative reconstruction and recognition theory

The method of normative reconstruction is embedded in Honneth’s general social philosophy. Fundamental to that philosophy is Hegel’s conception of freedom as social, i.e., as constituted through mutual recognition. In order to achieve self-realization and develop a positive identity it is essential for persons to be recognized by other persons and in particular by societal institutions (like the family, the economy and the state). To recognize individuals or groups specifically means “to ascribe to them some *positive* status” (Honneth, 1995, p. viii, italics his).

Honneth (1995, 2007b) has broadly distinguished three patterns of recognition that are important for self-realization and positive identity development: love, rights and solidarity. For love it is important that person’s affective and physical needs are met by proximal significant others (family, close friends), which aids the development of their basic self-confidence and self-worth. Misrecognition here comes from contempt, the violation of physical and psychological integrity in primary relationships. For rights it is important that persons are seen as morally responsible agents and bearers of equal legal, social and political rights, which aids the development of their self-respect. Misrecognition here comes from structural exclusion, the denial of equal rights to fully participate in and be respected as a member of society. For solidarity it is important that traits and abilities of persons are positively perceived and evaluated, which aids the development of their self-esteem. Misrecognition here comes from the denial or depreciation of a person’s contribution to a group, the economy or society, based on that person’s traits, convictions, ways of life, and other attributes.

From these patterns of recognition, Honneth (1995, 2007b) has deduced moral obligations that give rise to legitimate normative expectations of individuals or groups in society to be treated in accordance with these obligations and not to be misrecognized. A lack or negative forms of recognition that are subjectively experienced or perceived by individuals or groups may lead to struggles for more and/or for positive recognition, which should lead to the expansion or adaptation of existing patterns of recognition so as to fill the recognitive void.

While in this way often characterized as a “positive” or “perfectionist” approach (Lepold, 2019; McQueen, 2015), Honneth has been mindful of false and equivocal forms of recognition, amounting to “recognition as ideology” (Honneth, 2004, 2007a). Examples of the latter include praising Uncle Tom for his “submissive virtues,” imploring women to be “good” mothers and housewives, and “employee recognition programs” that only value employee behavior specifically geared at organizational performance targets (Hancock, 2024; Honneth, 2007a; Maia and Cal, 2014). All these provide forms of recognition that at the same time “bear features of domination” and in that way are incompatible with the “critical impulse” of recognition theory (Honneth, 2007a, p. 327). A solution to this problem can only be found in:

“the attempt to dissect and spell out the conditions under which forms of recognition are applied, such that the “irrational kernel” of all merely “ideological” forms of recognition will be revealed … This irrationality does not lie on the semantic surface of our evaluative vocabulary but is to be found instead in the discrepancy between evaluative promises and material fulfillment… Something in the physical world—be it modes of conduct or institutional circumstances—must change if the addressee or addressees are to be convinced that they have been recognized in a new manner” (Honneth, 2007a, pp. 327–328, 345).

Thus, at the institutional levels of society, normative expectations and evaluative promises of recognition have to be supported (or at least not contradicted) by other practices in such a way that these expectations and promises stand a good chance of being realized.

In order to ascertain this, Honneth has devised a method of normative reconstruction, in which these normative expectations and evaluative promises act as normative points of reference that are directly coupled to an analysis of their social reproduction in society (Honneth, 2014). For Honneth, this method starts by reconstructing:

“the normative promises which have made the different institutions of modern societies legitimate in the eyes of the participants; and then, “reconstruct” the conflictual actions and revolts by which they have tried … to bring about conditions which could help to realize these promises more sufficiently or adequately. By this reconstruction we can learn what those social conditions under which the normative principles of the different institutions could be realized would have to look like” (Curty, 2020, p. 1341).

As such, it is an immanent method; rather than confronting institutions and practices with external, abstract normative principles (like, for example, in Kantian or Rawlsian theories of justice), “the same standards according to which these institutions and practices are picked out of the chaos of social reality are used to criticize insufficient, still imperfect embodiments of… values” (Honneth, 2014, p. 9). By tapping into this “untapped normative surplus” of institutions (Zurn, 2015, p. 65), normative reconstruction becomes “critical,” in ways well comparable to what Horkheimer envisioned eight decades ago.

Normative reconstruction as an immanent method involves two steps. In the *first step*, general norms and values governing recognition in a certain institutional domain of society (e.g., family, economy, state) are detected, together with the more specific promises that accrue from them. In the *second step*, institutional practices that differentially affect the realization of these norms, values and promises in that domain are identified. When the norms and values governing recognition are sufficiently realized through institutional practices, Honneth labels this as “moral progress” (Curty, 2020, p. 1341). When institutional practices are seen as hindering or even preventing the realization of these norms, Honneth labels this as “normative misdevelopments” or even “normative paradoxes,” whereby a “contradiction is paradoxical when, precisely through the attempt to realize such a [normative] intention, the probability of realizing it is decreased” (Hartmann and Honneth, 2006, p. 47; Sutterlüty, 2017). This would occur, for example, when moral progress for one societal group has direct negative consequences for other groups, or when norms and values are realized in such a way that the particular content of these norms becomes lost or even converted into its opposite (Honneth and Sutterlüty, 2011).

The two steps are discernible in Honneth’s normative reconstruction of socioeconomic developments in Western capitalist countries after 1945 (Honneth and Sutterlüty, 2011). In the first step, he has described the rise of neoliberal capitalism in the 1980s (after three post-war decades of state-regulated capitalism) and its promises and expectations of entrepreneurialism, flexibility and involvement for employees in the “new spirit of capitalism” (Boltanski and Chiapello, 2018; Dorschel, 2022). In the second step, Honneth has analyzed how these promises and expectations are not supported and even contradicted by material conditions and institutional practices. Organizations only value entrepreneurialism within the strict confines of organizational goals, management control and performance measures, leading to “organized self-realization” as unfulfilled promises of authenticity, initiative and involvement to employees. Honneth has labelled all this as “paradoxes of capitalism” (Hartmann and Honneth, 2006; Honneth, 2004, 2007a). Such misdevelopments and paradoxes seem particularly prevalent in the platform economy and organizations partaking therein (like Uber, DPD UK and Amazon Flex), with platform organizations promising flexibility, (informational) self-determination and personal autonomy while at the same time creating working conditions and privacy breaches that undercut these promises (Helm and Seubert, 2020; Visser and Arnold, 2022).

In a related critique, Honneth (2008) has pointed at the important role of job interviews, where increasingly job candidates are expected to “sell” themselves, i.e., to provide compelling images and projections about how they envision their future job accomplishment, instead of reporting on past qualifications. Normative expectations of being recognized as an authentic individual with personal achievements are contravened here in two ways. First, it forces job candidates into defining their job-related attitudes, goals and wishes as “commodities” that can be manipulated at will, in a process that can be called self-reification (“Selbstverdinglichung”) (Honneth, 2008; Islam, 2012; Petersen and Willig, 2004). Second, the normative promise underlying this “selling of the self,” i.e. that individual authenticity could and should be meaningfully brought to the job, is violated by the fact that organizations do not value those forms of authenticity that do not fit their job descriptions or organizational values. It thus amounts to a “be spontaneous” paradox: be authentic, but in such a way as the organization expects of you (Voswinkel, 2011).

The latter examples already have brought normative reconstruction within the realm of management and organization (Honneth, 2010a, 2023; Smith, 2009). In order to illustrate its potential for critical analysis, in the next section the method of normative reconstruction is applied to two case examples from the literature. Both are illustrations from a “distant outsider” perspective, based on existing published research.

## 3 Two case examples

The first case involves an inductive, qualitative case study of 31 Uber drivers in Toronto, Canada, which examined their motives for driving for Uber and their lived experiences in doing so (Pettica-Harris et al., 2020). Normatively reconstructing the case study, in the first step it shows how Uber espouses values of freedom, flexibility, opportunity and possibility, certainly in comparison to more conventional taxi companies. Uber promises to be a comparably “*safer* place to pause from economic hardship and transitions, appealing to different types of drivers by supporting their unique and difficult personal circumstances” (Pettica-Harris et al., 2020, p. 52). In the second step, it shows that only to some extent Uber backs up these values and promises with actual material practices. The Uber drivers appreciate the flexibility and ease of signing up and feel that they are in the “driver’s seat,” both literally and figuratively. The control of their work through customer “star” ratings works both ways, since it also allows the drivers to provide feedback to each other about specific customers. At the same time, Uber charges high fees on the drivers’ fare, and in general the drivers do not see Uber as a lucrative source of income, given the long driving hours and the absence (at the time and location of the study) of career possibilities, retirement plans, training and development, and basic insurance coverage. Although then in a precarious position, the amount of suffering by the Uber drivers is mitigated by their tempered expectations: Uber just has to be “good enough” when compared to the alternatives (i.e., being unemployed or driving a regular taxi), and none of the drivers foresees a long-term career with Uber (Pettica-Harris et al., 2020, p. 53). In Honneth’s terms, there seems to be a normative misdevelopment involved, because ultimately Uber’s values and promises are not realized to a sufficient degree.

The second case involves a study, analyzing 430 self-reflection reports, written by freshmen trainee accountants during an annual summer course at a Dutch university. This study aimed to investigate the challenges and problematic situations these trainee accountants encountered during their first year at work in accounting firms (De Vries et al., 2022). Normatively reconstructing this study, in the first step it shows how these accounting firms espouse values of bottom-up socialization of trainees in their corporate communications, marketing and recruitment events, promising open and constructive dialogues and increasing recognition and understanding between supervisors and trainees as a two-way process. In the second step, it shows that the accounting firms on the whole do not back up these values and promises with actual material practices. As experienced by the trainees, socialization in the accounting firms is primarily top-down, in which the juniors are disciplined with values like “working overtime, accepting that they are in competition with peers for job security and placing a focus on obtaining commercial results on top of ensuring audit quality” (De Vries et al., 2022, p. 18). This is overlaid by a context of precarity: all trainees receive only temporary contracts and every year it is decided whether they are “up or out”—in other words, meet the standards for continuing employment or not. There is social and emotional hardship involved in this gap between trainees’ expectations, based on accounting firms’ espoused values, and the reality of work at these firms: “more than half of the new recruits… experienced suffering during their first year in an accounting firm. Moments of despair, fatigue, self-blame, frustration and exhaustion ran through almost all of our examples, and often appearing in conjunction” (De Vries et al., 2022, pp. 2–3). In Honneth’s terms, there seems to be a normative paradox involved, because the accounting firms’ material practices actually work to undermine their evaluative promises.

The normative reconstruction of both cases clearly brings out “ideological recognition,” as a form of recognition that holds a discrepancy between evaluative promises and their material fulfillment (Honneth, 2007a). In the neoliberal work practices involved, employees are often promised or told that they can or should be entrepreneurial, flexible and authentic, but at the same time they are often controlled and micro-managed and forced to adapt to the prevailing organizational culture instead. If on top of that their material (contractual and financial) working conditions are precarious, then they may suffer doubly: not only in a direct material sense, but also in the sense of being promised something that is directly contradicted by actual working conditions.

Cooper (2015, p. 14) captures broadly how work under such conditions evokes suffering comparable to a “living death,” following Cederström and Fleming (2012):

“The majority work for longer hours, less pay, fewer benefits, less security, and less promise of retirement and upward mobility…. In a sense, life itself has been put to work, i.e. our sociality, imagination, resourcefulness, and our desire to learn and share ideas. Corporations increasingly strive to harness these very human characteristics to drive value but neoliberal subjects have a hyper individualized expectation placed upon them to maximize returns on themselves. In practice, the majority have both a boss who gives orders, and an overwhelming management control system to deal with. It could be that this dual (and contradictory) pressure, to be entrepreneurial while also being closely controlled, is at least part of the reason behind the anguish suffered across all organizational levels… This is overlaid by the material conditions of exploitation, performance related pay, zero hour contracts, falling real wages, the removal of social security safety nets etc.”

## 4 Conclusion and discussion

In this paper Honneth’s recognition theory and method of normative reconstruction has been proposed as a possible new approach to CMS’s relations to organizational practice and practitioners and illustrated with two cases from the literature. While in this way seemingly applicable to organizational analysis and reflecting an increasing popularity of Honneth in MOS more in general, two points of discussion are in order here.

As a first point of discussion, it has been argued that Honneth, especially in his magnum opus *Freedom’s Right* (2014), presents an overly idealized picture of social institutions as rational forms reproducing social freedom (Ng, 2019; Shafer, 2018). The recognitive norms and values that Honneth sees immanently present in different spheres of society in the form of “normative surpluses” (Hartmann and Honneth, 2006; Zurn, 2015) represent a too optimistic picture of current society. This holds especially for the market sphere, characterized by a neoliberal capitalism intent on subverting these “normative surpluses,” and thus depriving Honneth’s approach of both its normative and critical edge and its sociological dimension (Carleheden, 2021; Fazio, 2019; Johnson, 2014; Jütten, 2015). Honneth himself has expressed surprise and pessimism about the enduring success of neoliberalism and the ideological and ethical ideals with which it appears to attract people, in spite of recurring global financial crises. As a possible explanation he has pointed at the current weakness of unions vis-à-vis economic globalization (Honneth, 2019; Marcelo, 2013).

In response, Honneth’s method may be supplemented with the work of the French pragmatist sociologists Boltanski and Chiapello (2018), aided by the fact that Honneth has positively related his work to French pragmatic sociology in general (Boltanski et al., 2014; Honneth, 2010b), and in his “paradoxes of capitalism” papers regularly refers to it as an important source of inspiration for his critique of entrepreneurialism (Hartmann and Honneth, 2006; Honneth, 2004, 2007a; Honneth and Sutterlüty, 2011).

Put briefly, Boltanski and Chiapello (2018) have observed that capitalism at its core is a system of unlimited capital accumulation that by itself is unattractive for both wage earners and for capital owners. Such a system is always in need of an ideology, or “spirit of capitalism,” that serves to justify people’s engagement with it and which renders this engagement attractive. More than to its proponents, for these forms (or “regimes”) of justification capitalism typically turns to its enemies, because “these are the people who provide it with the moral foundations that it lacks, and who enable it to incorporate justice-enhancing mechanisms whose relevancy it would not otherwise have to acknowledge” (Boltanski and Chiapello, 2018, pp. 27–28).

Regimes of justification offer concrete ideas for everyday discourse and practice in dealing with capital accumulation by indicating, first, what is exciting about capitalism (i.e., how people can become enthusiastic about it, although not many are able to fully share in its profits), second, how capitalism provides a minimum of security (i.e., how those people not fully sharing in its profits nevertheless are protected and cared for), and third, how capitalism is consistent with notions of fairness (i.e., how it is imbued with a sense of justice and contributes to the common good) (Boltanski and Chiapello, 2018, p. 16; Chiapello, 2013).

Supplementing Honneth’s method of normative reconstruction with the concept of regime of justification addresses this first point of discussion in two ways. First, it reconfirms an immanent approach by pointing out that there *always* will be norms, values and promises with which the capitalism system and its institutions will attempt to appear exciting, attractive and fair to the people, which norms and values then can be put to the test of normative reconstruction. Second, it enables a redirection of research attention from the more abstract level of spheres and institutions to the more concrete levels of sectors, subsystems and organizations, for example within the market sphere, where regimes of justification (visible in corporate and marketing communications toward employees, customers and other stakeholders) exist alongside capitalist practices of guarding the financial “bottom line” in the service of unlimited capital accumulation (Visser and Arnold, 2022).

As a second point of discussion, the method of normative reconstruction may be helpful in connecting different forms of CMS scholars’ engagement with practice by instilling a uniform pattern of inquiry at various levels of engagement. Whether applied at the macro-level of Western societies (e.g., Hartmann and Honneth, 2006; Honneth, 2014) or at the more micro-level of organizations (e.g., Honneth, 2010a, 2023), the purpose of normative reconstruction always is, first, to detect general norms and values governing recognition in a certain institutional domain or organization, together with the more specific promises that accrue from them and, second, to identify the institutional practices that differentially affect the realization of these norms, values and promises in that domain or organization.

When applied at the macro-and meso-levels, normative reconstruction involves a “distant outsider” position, based on the CMS researcher’s reading and interpretation of existing published research, data and internet sources. Critique may then be published via books, articles, lectures and social media, hoping to mobilize public opinion and consumer consciousness against unsustainable and unjust corporate practices (Alvesson, 2021; Fleming and Banerjee, 2016).

When applied at the more micro-level of organizations, a “reflective insider” position becomes possible with a dialogical notion of normative reconstruction as “an interactive exchange where both observer and participant look for a deeper and more complex understanding of the contradictions of social reality, its emancipatory ideals and their own practical role within it,” whereby outsider and insider positions also may shift in the course of an inquiry (Texeira, 2017, p. 608; Alvesson and Spicer, 2025).

Honneth’s approach is thus geared toward all institutional and organizational situations in which the realization of general norms, values and promises governing recognition is differentially affected by concrete practices in such a way that false, ideological forms of recognition obtain. Pointing out the pervasive discrepancies between espoused and factual recognitive norms and values gives a critical edge to the analysis of these institutional and organizational situations, sharpening awareness of ambiguities and contradictions therein.

In principle, this method of normative reconstruction may be extended to other organizational phenomena where “discrepancies between evaluative promises and material fulfillment” may be reasonably expected. One example is corporate social responsibility, according to which corporations promise to be socially and ecologically responsible beyond the financial “bottom line,” but which promise they often fail to materially fulfill by evading taxes, environmental extraction and pollution and maintaining sub-standard working conditions (Schneider, 2020; Sikka, 2010). Another example is diversity and inclusion, according to which organizations promise policies aimed at diminishing gender, ethnic and class differences among their personnel, but which promise they often only maintain as long as it does not jeopardize the financial “bottom line” and the masculine working culture conducive to it (Benschop, 2021; Fraser, 2009).

All in all, the conclusion is that Honneth’s recognition theory and method of normative reconstruction have the potential to contribute positively to existing CMS theory and engagement with practice. Method and theory provide a powerful way to critically assess various forms of corporate double talk, ambiguity and sanctimony. In that way, Honneth’s approach contributes to the realization of humanistic values in organizations as they take up their pivotal role in the major global challenges of today.
